# Klüver–Bucy syndrome secondary to a nondominant middle cerebral artery ischemic stroke: a case report and review of the literature

**DOI:** 10.1186/s13256-021-02932-0

**Published:** 2021-07-15

**Authors:** Alawi Aqel Al-Attas, Abdulrahman Yousef Aldayel, Tareq Hamad Aloufi, Nabil Biary

**Affiliations:** Department of Neurology, Prince Sultan Military Medical City (PSMMC), MakkahAl Mukarramah Road, As Sulimaniyah, PO Box 7897, Riyadh, Saudi Arabia

**Keywords:** Klüver–Bucy syndrome, Stroke, Case report, Ischemic stroke, Neurobehavioral symptoms, Temporal lobe damage

## Abstract

**Background:**

Klüver–Bucy syndrome is a rare and complex neurobehavioral cluster that occurs in humans and results from a temporal lobe lesion. It can be associated with a variety of causes. Stroke is a rarely reported cause of this syndrome.

**Case presentation:**

In this report, we present the case of a 68-year-old Saudi male who developed Klüver–Bucy syndrome subsequent to a nondominant middle cerebral artery ischemic stroke involving right temporal lobe. The patient manifested most of the Klüver–Bucy syndrome clinical features, including hypersexuality, hyperphagia, hyperorality, and visual hypermetamorphosis (excessive tendency to react to every visual stimulation with a tendency to touch every such stimulus). These neurobehavioral manifestations improved after he was started on treatment.

**Conclusions:**

The clinical course, anatomical association relying on pathophysiology, and potential treatment have all been deliberated in regard to the rare occurrence of Klüver–Bucy syndrome resulting from temporal lobe pathology.

## Background

Klüver–Bucy syndrome (KBS) is a very rare and peculiar clinical entity. It is the consequence of bilateral temporal lobe lesions, particularly involving the amygdala and hippocampus [1]. In 1937, Heinrich Klüver and Paul Bucy first defined a neurobehavioral syndrome in rhesus monkeys after bilateral temporal lobectomies [2, 3]. This syndrome featured hyperorality, hypersexuality, placidity, hyperphagia, visual agnosia, and altered emotional behavior [2, 4]. Patients often present with only three or more symptoms and are designated as partial KBS [5]. The causes of KBS vary among the previously reported cases. These include herpes simplex encephalitis (HSE), Alzheimer’s disease, Niemann–Pick disease, stroke, Huntington’s disease, traumatic brain injury, toxoplasmosis, shigellosis, hypoglycemia, juvenile neuronal lipofuscinosis, acute intermittent porphyria, and tubercular meningitis [4, 6]. KBS is diagnosed clinically by the presence of its characteristic symptoms. Brain magnetic resonance imaging (MRI) is used to confirm the diagnosis by demonstrating bilateral temporal lobe mutilation [4]. Furthermore, appropriate assessment to identify the underlying etiology is part of the general management of KBS [4]. The treatment of KBS is mainly supportive measures, including occupational therapy, selective serotonin reuptake inhibitors for behavioral changes, and carbamazepine for hypersexuality [4, 7]. The first KSB case was reported in 1975. It was a 22-year-old man with bilateral temporal lobe damage as a result of HSE [8]. In the literature, there are limited contemporary published cases of KSB secondary to stroke [9–11]. In this case report, we present an unusual case of an elderly male who developed KBS features following an ischemic stroke in his nondominant hemisphere.

## Case presentation

A 68-year-old Saudi male known to have type 2 diabetes mellitus and hypertension for 15 years presented to the emergency department of the Prince Sultan Military Medical City, Riyadh, Saudi Arabia, in September 16, 2018 with confusion, vomiting, left-sided weakness, left facial palsy, and slurred speech. His symptoms had started 2 hours and 25 minutes earlier.

**Fig.1:**
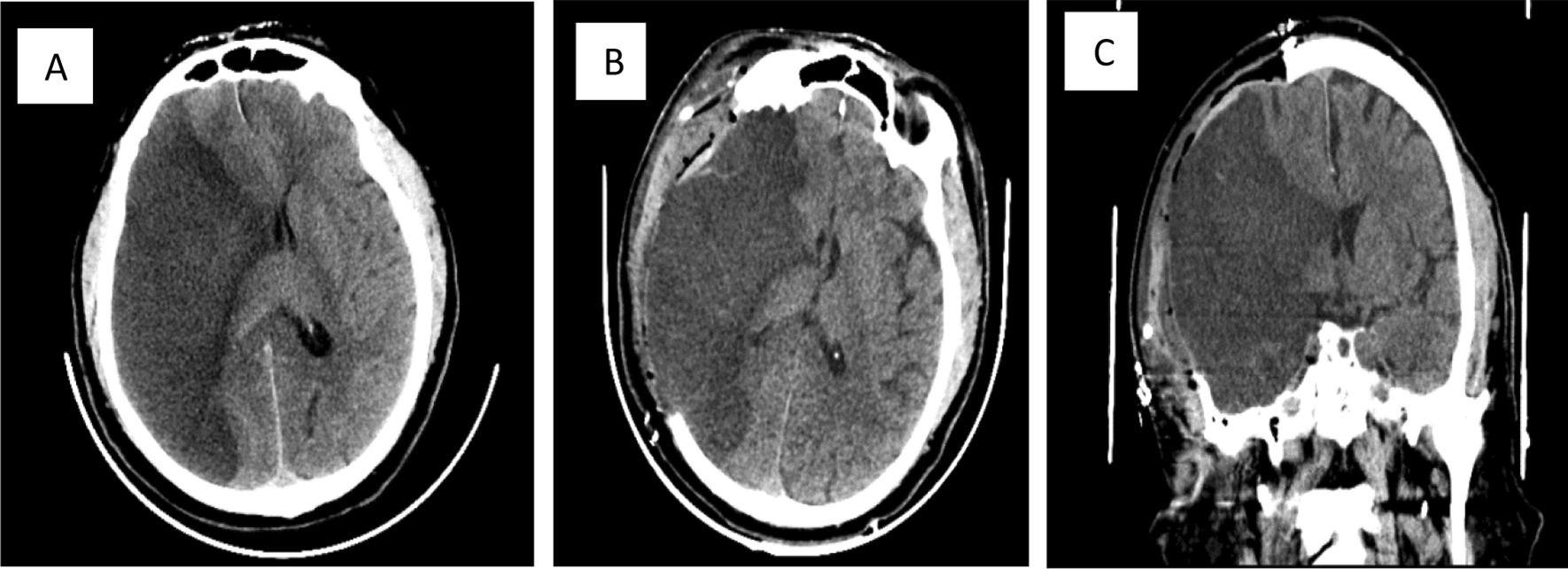
**A** Extensive hypodense lesion on the right temporal area on the axial noncontrast brain CT before thrombectomy. **B** Large hypodense lesion of the right temporal area after craniotomy was done on the axial noncontrast brain CT. **C** Coronal brain largely affected by hypodense lesion on the right temporal area

**Fig. 2:**
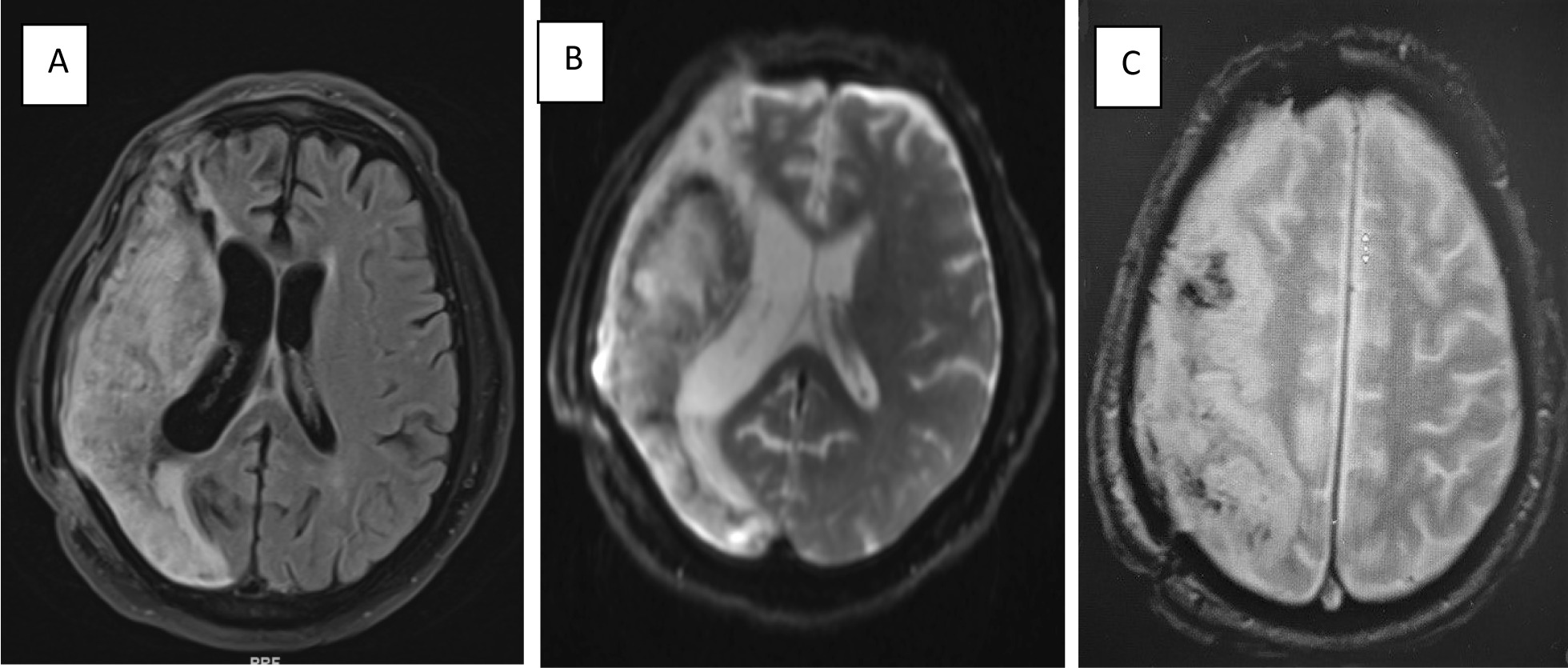
**A** Axial section of brain MRI Fluid-Attenuated Inversion Recovery (FLAIR) image showing subacute infarction extensively affecting the right temporal area. **B** Lesion in the T2-weighted MRI image. **C** Gradient echo showing blooming artifacts representing hemorrhagic transformation

On examination, the patient was conscious but confused. He had left facial weakness, gaze deviation to the right side, left homonymous hemianopsia, dense left hemiplegia, and left sided hemineglect, so the stroke code was announced. Both a computed tomography (CT) scan and a CT angiogram were performed upon admission. The CT scan of the brain showed an ischemic stroke of the right temporal lobe surrounded by diffuse brain edema causing mass effect (Fig. 1). The CT angiogram revealed nonopacification of the right internal carotid artery near its origin with extension cranially to involve the majority of the cervical portion in addition to the petrous, cavernous, and supraclinoid aspects. In addition, there was nonopacification of the right middle cerebral artery (MCA) branches, which was likely due to thrombus formation. Additionally, the brain MRI findings are illustrated in Fig. 2.

The patient was given tissue plasminogen activator (tPA) as per the stroke protocol. Later on, he was taken for intraarterial thrombectomy, which, after five attempts, was without significant reperfusion. He was then intubated during the procedure, and later on transferred to the intensive care unit for postthrombolytic protocol. A follow-up CT scan showed a malignant MCA stroke with diffuse edema causing mass effect, midline shift, and impending herniation. Therefore, the patient was taken for craniotomy as the only salvageable option, and high intracranial pressure measures were reversed. A few days later, the edema improved, the mass effect resolved, and the patient was weaned off sedation. Afterward, the patient started to slowly recover.

Several laboratory investigations were conducted, including complete blood count, coagulation profile, and electrolyte panel. Markers of vasculitis and autoimmune disorders were unremarkable. Hemoglobin A1C was 10.2%. The lipid profile was the following: cholesterol: 4.73 mmol/L, triglycerides: 2.07 mmol/L, high-density lipoprotein (HDL): 1.05 mmol/L, and low-density lipoprotein (LDL): 3.25 mmol/L.

On November 19, 2018, the patient was transferred to the neurology ward. A few days later, it was noticed that he was displaying hyperorality, hypermetamorphosis, hyperphagia, hypersexuality, and other behavioral changes, including apathy, visual agnosia, and hallucinations. During the first few days in the ward, the patient was unable to recognize his family members. Hence, he was started on escitalopram 10 mg once daily together with levetiracetam 1 g twice daily. Two months later, the patient showed significant improvement in his neurobehavioral symptoms, hypersexuality, and altered eating behavior.

## Discussion

To the best of our knowledge, this is the first reported case of KBS in Saudi Arabia as a result of a unilateral ischemic stroke of the nondominant temporal lobe. Any combination of three or more clinical manifestations is suggestive of partial KBS. It is very rare to have complete KBS in humans [12–14], and it usually exists incompletely [15]. In humans, it is commonly accompanied by apathy and amnesia, which was observed in our case. Although in most cases KBS has been observed following bilateral lesions of the temporal lobe, some cases without bitemporal impairments have been reported [16–18]. The most important clinical manifestations observed in our case were altered sexual conduct, increased dietary intake, apathy, hyperorality of nearby items (including inedible and sharp objects), and visual hypermetamorphosis. This accounts for five of the major features of KBS, as reported by Rossitch, Oakes, Hreniuc *et al.* in their previously published studies [10, 18].

In our case, hyperorality was observed as grasping with the hands only. A case reported by Pilleri disclosed that oral grasping was associated with lesions of the temporal lobe, while grasping with the hands was associated with lesions of the frontal lobe [19]. Our patient demonstrated visual hypermetamorphosis, which is defined as an irresistible impulse to notice and react to anything within sight. The alteration of sexual conduct was apparent as physical hypersexuality, as observed by Chou *et al.*, whereas the hypersexuality of our patient was emotional. The increased libido seen in KBS has been attributed to the loss of limbic system cortical inhibitory tracts [20]. Both the frontal lobe and the limbic system have projections that come from the temporal cortex, amygdala, and thalamus. These structures are known to be related to emotions [21]. In a previous study, the manifestation of KBS was linked to a good prognosis in the clinical outcome of a cerebral insult associated with loss of consciousness [22].

Anatomically, KBS is frequently due to lesions affecting bilateral temporal lobes. In addition, the hippocampus, uncus, cingulate gyrus, and amygdala play a significant role in the pathogenesis of KBS. Moreover, a disturbance of the pathways that provide the neurological connections of the limbic system, medial thalami, and frontal lobe is accountable for impaired memory and emotions, which also contribute to the development of KBS [23, 24]. In other cases, KBS symptoms were found after unilateral brain lesions, such as right subdural hematoma, infarction, or lobectomy of the left temporal lobe. In previous published studies, this syndrome is a consequence of visual input interruption to the limbic system as described by Geschwind [25]. Müller *et al.* mentioned that KBS seems to follow an obliteration of the thalamus, frontal cortex, and limbic system pathway connections [17], and Hreniuc *et al.* stated that their patient has disruption in the right temporal lobe and amygdala. However, in our case, the patient has a lesion in the temporal area of the nondominant hemisphere; this has been reported by Hreniuc *et al.* in their patient [10]. Since a disturbance in the level of consciousness at the time of recovery might account for the manifestations of KBS, the CT scan is a distinguishing imaging test for patients who suffer brain injuries. Additionally, for those who present with neurobehavioral symptoms, MRI is necessary to structurally visualize brain lesions [23, 26]. The clinical manifestations of this syndrome may appear in patients without morphological changes in the brain CT scan, particularly the temporal lobes. For this reason, brain evaluation by MRI scan should be considered in patients with cerebral insults.

The clinical results of KBS are distinctive from one case to another. It is usually reversible in patients with traumatic injury, seizures of any cause, or infection if undergoing prompt diagnosis and early onset of treatment. The point that precocious cognition and implementation of the treatment plan for the drastic behavior changes requiring rehabilitation can be avoided has been entailed in the various courses of KBS [9]. The effect of treatment varied among KBS patients. Some cases showed dramatic improvements when treated with antiepileptics, such as carbamazepine, or serotonin reuptake inhibitors [4, 27]. Other cases showed persistent mental derangements, and others may develop Korsakoff syndrome [28].

## Conclusions

KBS is rarely caused by an ischemic stroke, especially in the nondominant hemisphere. KBS, as illustrated in our case as a consequence of an ischemic stroke of the right temporal lobe, is supported by other previously reported studies, and it can impart distinct insight into the possible pathophysiology of KBS. Further studies are needed to elucidate the pathophysiology of this disease and, hence, help explicitly formulate potentially effective treatments.

## Data Availability

All data and materials are available upon request from the corresponding author.

## Abbreviations

CT: Computed tomography
HDL: High-density lipoprotein
HSE: Herpes simplex encephalitis
KBS: Klüver–Bucy syndrome
LDL: Low-density lipoprotein
MCA: Middle cerebral artery
MRI: Magnetic resonance imaging
tPA: Tissue plasminogen activator
